# Exploration of candidate genes associated with rare SNVs in pulmonary stenosis using whole-exome sequencing and machine learning

**DOI:** 10.3389/fgene.2026.1765512

**Published:** 2026-06-05

**Authors:** Yuting Liu, Sun Chen, Yongzhou Liang, Suqiu Huang, Bingyao Zhang, Shuqi Liu, Ling Yang, Liqing Zhao, Rang Xu, Yurong Wu

**Affiliations:** 1 Department of Pediatric Cardiology, Xinhua Hospital, Shanghai Jiao Tong University School of Medicine, Shanghai, China; 2 Department of Women’s and Children’s Health Care, Shanghai Key Laboratory of Maternal Fetal Medicine, Shanghai Institute of Maternal-Fetal Medicine and Gynecologic Oncology, Shanghai First Maternity and Infant Hospital, School of Medicine, Tongji University, Shanghai, China; 3 Department of Pediatrics, West China School of Medicine, Sichuan University, Sichuan University Affiliated Chengdu Second People’s Hospital, Chengdu, Sichuan, China; 4 Department of Cardiology, Shanghai Children’s Hospital, Shanghai Jiao Tong University School of Medicine, Shanghai, China; 5 Scientific Research Center, Xinhua Hospital, Shanghai Jiao Tong University School of Medicine, Shanghai, China

**Keywords:** congenital heart disease, machine learning, pulmonary stenosis, rare variant, whole-exome sequencing

## Abstract

**Background:**

Pulmonary stenosis (PS) is a common form of congenital heart disease (CHD) that impairs cardiopulmonary function and can be life-threatening in severe cases. As a complex polygenic disorder, the genetic basis of PS remains incompletely understood.

**Methods:**

Rare pathogenic single nucleotide variants (SNVs) were identified from whole-exome sequencing (WES) data of 185 sporadic PS patients and 100 healthy controls using multiple pathogenicity-filtering strategies. Gene-level burden test was performed, with complementary analysis using sequence kernel association test–optimal (SKAT-O). Three machine learning algorithms—least absolute shrinkage and selection operator (LASSO), random forest (RF), and extreme gradient boosting (XGBoost)—were applied to prioritize candidate genes. The overlap between machine learning–based selections and burden test results was systematically evaluated. Final candidate genes were further prioritized through protein–protein interaction (PPI) network analysis, and their expression in human pulmonary artery endothelial cells (HPAECs) was assessed by reverse transcription quantitative polymerase chain reaction (RT-qPCR).

**Results:**

Comparative analyses showed that different machine learning algorithms exhibited distinct feature selection patterns, with RF demonstrating the highest concordance with burden test results. A total of 17 candidate genes were prioritized (*HAND2, SETD2, KDM6B, NCOR2, FLNB, NOTCH3, DNAH5, PLEC, COL5A1, KIF7, CLTCL1, XRN1, ITPR2, SCRIB, PYGB, IQGAP3, and SHC2*).

**Conclusion:**

These findings indicate that machine learning can complement conventional gene-based analyses of WES data. This study provides a set of candidate genes associated with PS and offers a basis for further investigation of its genetic architecture.

## 1 Introduction

Congenital heart disease (CHD) affects approximately 1 in every 100 live births, with pulmonary stenosis (PS) accounting for 8%–12% of cases, making it one of the most common subtypes (Weaver et al., 2022). The pulmonary valve forms the outflow tract of the right ventricle. Structural abnormalities of this valve lead to right ventricular outflow tract obstruction, resulting in right ventricular hypertrophy and reduced compliance, which can impair both systolic and diastolic function.

Pulmonary valve development is initiated after rightward looping of the cardiac tube, during which endocardial cushions form within the outflow tract. A subset of endothelial cells undergoes endothelial-to-mesenchymal transition (EMT), followed by migration and proliferation within the cardiac jelly to form mesenchymal cells. This process is regulated by multiple signaling pathways, including bone morphogenetic protein (BMP), transforming growth factor-β (TGF-β), NOTCH, and WNT (Garside et al., 2013; Henderson et al., 2021; Ahmed et al., 2023). As development progresses, the cushions remodel into valve leaflets composed of endothelial layers and extracellular matrix components.

From a genetic perspective, PS is considered a complex polygenic disorder with an incompletely defined genetic basis. It is observed in several syndromic conditions, including 3q29 microdeletion syndrome (Kaba and Çelik, 2022), Noonan syndrome (NS) (Sun et al., 2022), and LEOPARD syndrome (Galazka et al., 2020). In sporadic cases, whole-exome sequencing (WES) has been widely applied. Previous WES studies have identified candidate genes in PS with pulmonary atresia and intact ventricular septum (PS-PA/IVS), including APC, PPP1R12A, and PCK2 (Zhou et al., 2021). However, large-scale WES studies focusing specifically on isolated PS remain limited.

In this study, WES data from 185 sporadic PS patients and 100 healthy controls were analyzed using a gene prioritization framework. Gene-level burden test was performed and integrated with three machine learning algorithms to rank candidate genes. Based on this approach, 17 candidate genes were prioritized. These results are presented as preliminary findings.

## 2 Methods

### 2.1 Case ascertainment

Medical records, imaging data, and surgical reports of 185 Asian pediatric patients with sporadic PS were retrospectively reviewed at Xinhua Hospital, Shanghai Jiao Tong University School of Medicine, between January 2020 and June 2024. All diagnoses were confirmed by echocardiography and/or surgical findings. Doppler echocardiography was used as the primary diagnostic modality. Based on peak transvalvular pressure gradient and flow velocity across the pulmonary valve, PS was classified into three severity grades: mild (peak gradient < 36 mmHg, peak velocity < 3 m/s), moderate (36–64 mmHg, 3–4 m/s), and severe (> 64 mmHg, > 4 m/s). Critical pulmonary stenosis (CPS) was defined by hypoplasia of the pulmonary valve annulus and right ventricle, frequently accompanied by a smaller tricuspid annulus, reduced right ventricular cavity size, and more severe tricuspid regurgitation. In addition, supravalvular or subvalvular PS was identified when obstruction was located above the pulmonary valve (main or branch pulmonary artery) or below the valve (right ventricular outflow tract). Based on imaging findings and flow velocity, patients were categorized into five groups: mild PS, moderate PS, severe PS, CPS, and PS with supravalvular or subvalvular stenosis. Patients with syndromic disorders or tetralogy of Fallot were excluded to ensure a relatively homogeneous cohort. Minor cardiac structural abnormalities other than PS were also recorded.

The control group included 100 Asian healthy children without documented developmental abnormalities who had previously undergone whole-exome sequencing (WES) (Liu et al., 2022). Peripheral blood samples were collected from cases and controls after obtaining written informed consent from participants and/or their legal guardians. The study was approved by the Ethics Committee of Xinhua Hospital, Shanghai Jiao Tong University School of Medicine (XHEC-C-2012-018).

### 2.2 DNA extraction and WES

Peripheral blood (1–2 mL) was collected in EDTA-anticoagulated tubes. Genomic DNA was extracted using the DNeasy Blood and Tissue Kit (Qiagen) according to the manufacturer’s instructions. Target enrichment was performed using the Agilent SureSelect Target Enrichment Kit (V8 + CREV4) for cases and version V5 for controls. Sequencing was conducted on the Illumina NovaSeq platform to generate 150 bp paired-end reads. Bioinformatic processing and variant annotation were performed as previously described (Fontebasso et al., 2013).

### 2.3 Variant quality control, alignment, and annotation

Raw sequencing data were processed using a standardized Genome Analysis Toolkit (GATK, version 4) pipeline (McKenna et al., 2010; DePristo et al., 2011) by Shanghai Jingzhou Gene Technology Co., Ltd. Data quality was assessed using FastQC (v0.11.7), and adapter trimming and low-quality reads were filtered using fastp (v0.12.5). Clean reads were aligned to the hg19 reference genome using Burrows–Wheeler Aligner (BWA-MEM, v0.7.17) (Li and Durbin, 2009). PCR duplicates were marked using Picard MarkDuplicates (v2.18.0). Base quality score recalibration (BQSR) was performed using GATK (v4.1.9.0). Variant calling for single nucleotide polymorphisms (SNPs) and insertions/deletions (indels) was conducted using HaplotypeCaller (Poplin et al., 2018), followed by joint genotyping with CombineGVCFs and GenotypeGVCFs. Variant quality score recalibration (VQSR) was applied using VariantRecalibrator and ApplyVQSR. Functional annotation was performed using ANNOVAR (2017 version) (Wang et al., 2010) following GATK Best Practices.

### 2.4 Initial variant filtering and probe set intersection

Different exome capture kits were used for cases (Agilent SureSelect V8 + CREV4) and controls (V5). To reduce potential batch effects arising from differences in probe design, the target regions of the two kits were intersected using BEDTools (Quinlan and Hall, 2010), and only shared regions were retained for downstream analyses.

Initial filtering of single nucleotide variants (SNVs) and insertions/deletions (indels) was performed using the following criteria:

1. Variants passing GATK quality filters;
2. Variants located in exonic or splicing regions, with intronic, untranslated regions (UTRs), and other noncoding variants excluded;
3. Removal of synonymous variants, retaining only missense and loss-of-function (LOF) variants, including frameshift, stop-gain, start-loss, and splice-site variants.

After preliminary filtering, all retained nonsynonymous exonic and splicing variants were restricted to the intersected probe regions shared by the V8+CREV4 and V5 capture kits to ensure analytical consistency. Indel detection remains more challenging than SNV detection due to factors such as read alignment complexity, variable capture efficiency, elevated error rates in microsatellite regions, and an increased propensity for false-positive calls in repetitive sequences (Narzisi et al., 2014). Benchmarking studies using the reference sample NA12878 have demonstrated that the V5 capture kit achieves a mean sensitivity of >99.4% for SNVs and >94% for indels, with corresponding false discovery rates of <0.2% and <1.5%, respectively (Gibson et al., 2018). Considering these technical limitations, and to improve analytical robustness and comparability between datasets, only SNVs were retained for subsequent variant filtering and case–control analyses.

### 2.5 Filtering of rare pathogenic variants

High-confidence rare pathogenic SNVs were identified using the following criteria:

1. Variants with depth of coverage (DP) < 30× or genotype quality (GQ) < 40 were excluded;
2. Variants with minor allele frequency (MAF) < 0.001 in public databases, including the 1000 Genomes Project, ExAC, gnomAD, ESP6500, and Kaviar, were retained; variants absent from all databases were also included;
3. Missense variants predicted to be damaging by at least one of the following tools were classified as deleterious missense variants: SIFT, PolyPhen-2, and MutationTaster.

Pseudogenes annotated in the Ensembl database were excluded from downstream analysis (Supplementary Table S1).

### 2.6 Gene burden test

Gene-level burden test was performed using 185 PS cases and 100 controls. Rare pathogenic variants were aggregated at the gene level. The odds ratio (OR) was calculated for each gene based on the number of individuals with and without variants in cases and controls. Genes with OR > 1 were retained for downstream analysis as a directional filtering step to focus on variants enriched in cases. This step was not used as a statistical significance criterion but to prioritize candidate genes for further evaluation. For each gene, a 2 × 2 contingency table (variant carrier status × case/control status) was constructed, and Fisher’s exact test was applied to assess association. P-values were adjusted for multiple testing. Due to limited statistical power for rare variants, no gene remained significant after correction. Therefore, genes with nominal P < 0.05 were retained as an exploratory filter.

### 2.7 Sequence kernel association test–optimal (SKAT-O) analysis

To complement the burden test, gene-level association testing was performed using the sequence kernel association test–optimal (SKAT-O) method implemented in the R package *SKAT* (Lee et al., 2012). The same rare pathogenic SNV matrix was used. A binary phenotype model (case/control) was applied, and gene-level associations were evaluated using the optimal unified test framework. Multiple testing correction was performed using the Benjamini–Hochberg method. Genes with nominal P < 0.05 were retained for comparison with burden test results.

### 2.8 Machine learning–assisted prioritization using multiple algorithms

Given the relatively relaxed threshold used in the burden test, multiple machine learning algorithms were incorporated to provide complementary evidence for gene prioritization. Considering PS as a complex polygenic disease, an additive genetic model was adopted, with genotypes coded as 0 (wild-type homozygous), 1 (heterozygous), and 2 (mutant homozygous). For all genes with OR > 1, an individual-level mutation burden matrix was constructed by summing the number of variants per gene. This matrix was annotated with case/control labels and used as input for downstream analyses.

In this study, machine learning approaches were applied for feature ranking and prioritization rather than predictive model construction. Accordingly, the full dataset was used to estimate feature importance, and stability was evaluated through cross-validation procedures and ensemble-based importance measures. Three supervised machine learning algorithms were applied to prioritize candidate genes.

Least absolute shrinkage and selection operator (LASSO) regression (Ranstam and Cook, 2018; Tibshirani, 2018; Yang et al., 2023), implemented using the “glmnet” package in R (version 4.2.2), applies an L1 regularization penalty for variable selection. A 10-fold cross-validation procedure was used to determine the optimal regularization parameter (λ). The λ value corresponding to the minimum cross-validation error (λ_min) was selected, and genes with non-zero coefficients were retained. Random forest (RF) (Yang et al., 2023; Guan et al., 2024), implemented using the “randomForest” package, is an ensemble learning method based on multiple decision trees. The initial model was constructed with 500 trees (ntree = 500), and the optimal number of trees was determined based on the minimum out-of-bag (OOB) classification error rate. The final model was rebuilt using this optimal tree number. Feature importance was calculated based on the mean decrease in Gini impurity, and genes were ranked accordingly. Extreme gradient boosting (XGBoost), implemented using the “xgboost” package through the “caret” framework, is a gradient boosting algorithm suitable for high-dimensional data. Model training was performed using repeated 10-fold cross-validation (four repeats). Feature importance was calculated based on gain, and genes were ranked accordingly.

### 2.9 Comparison of genes prioritized by burden test and machine learning algorithms

Genes from the burden test were ranked by ascending P-values. Genes identified by LASSO were ranked by the absolute values of regression coefficients, whereas genes from RF and XGBoost were ranked by their respective feature importance scores. The top 10 and top 20 genes from each machine learning algorithm, together with genes identified in the burden test at nominal thresholds (P < 0.01 and P < 0.05), were listed separately. Overlap between genes identified by the burden test and those prioritized by each machine learning algorithm was assessed to evaluate concordance among methods.

### 2.10 Selection of consensus candidate genes

Gene selection was based on the burden test in combination with results from the three machine learning algorithms. To ensure comparability across methods, the number of genes retained from each machine learning algorithm was matched to the number of genes identified in the burden test at nominal P < 0.05 (n = 142). From the 142 genes identified in the burden test, genes were selected as initial candidates if they met at least two of the following criteria:

1. Included among the 133 genes identified by LASSO;
2. Ranked within the top 142 genes by RF;
3. Ranked within the top 142 genes by XGBoost.

### 2.11 PS- and CHD-Associated susceptibility gene sets

The PS-associated gene set was obtained primarily from the CHDbase database (Zhou et al., 2023), which includes 1,124 CHD susceptibility genes curated from 1,114 publications. Genes associated with PS (from 137 publications) were extracted to generate the PS gene list. Additional PS-associated genes were retrieved from the DisGeNET database (Piñero et al., 2020) using the terms “Pulmonary Valve Stenosis (C0034089)” and “Congenital pulmonary valve stenosis (C0162164).” A filtering threshold of scoreGDA > 0.1 was applied. In total, 198 PS-associated genes were compiled for downstream analysis (Supplementary Table S2). A broader CHD-associated gene set was generated by combining CHDbase with a previously curated list of human CHD-related genes (Jin et al., 2017) (Supplementary Table S3).

### 2.12 Protein–protein interaction (PPI) network analysis

PPI analysis was performed using the STRING database (Szklarczyk et al., 2023). Initial candidate genes and known PS-associated genes were jointly analyzed to construct an interaction network. The network was visualized using Cytoscape (Shannon et al., 2003). PPI analysis was used as an exploratory approach to assess potential interactions among candidate genes. Initial candidate genes showing direct or indirect interactions with known PS-associated genes were retained for further analysis. Network centrality was evaluated using node degree, and the overall degree distribution was examined with reference to median and mean values. Based on the degree distribution, a cutoff of degree > 5 was applied. Genes interacting with at least six nodes were retained as candidate hub genes.

### 2.13 RNA extraction and RT-qPCR in HPAECs

Total RNA was extracted from HPAECs using TRIzol reagent. One microgram of RNA was reverse-transcribed into complementary DNA (cDNA) using the PrimeScript RT Reagent Kit (RR036A, TaKaRa). Quantitative PCR was performed using TB Green Premix Ex Taq (RR420A, TaKaRa) on a QuantStudio 3 Real-Time PCR System (Applied Biosystems, Thermo Fisher Scientific). GAPDH was used as the internal control. Primers were designed using Primer Premier 6 or obtained from previously published studies (Xu et al., 2019; Yang et al., 2020; Shen et al., 2021), and are listed in Supplementary Table S4.

### 2.14 Statistical analysis

All statistical analyses and data visualization were performed using R software (version 4.2.2) and GraphPad Prism (version 10.1.2). Gene-based association analyses were conducted using Fisher’s exact test and the SKAT-O method, with P values adjusted for multiple testing where applicable. Machine learning analyses were performed in R using appropriate packages as described above. Unless otherwise specified, two-tailed P values < 0.05 were considered statistically significant.

## 3 Results

### 3.1 Overview of clinical and baseline characteristics of the cohort

The study cohort included 185 sporadic, unrelated patients with a primary diagnosis of PS recruited from Xinhua Hospital, Shanghai Jiao Tong University School of Medicine, between January 2020 and June 2024 (n = 185; males = 95; females = 90; mean age = 1.25 ± 2.15 years). Based on transvalvular flow velocity and echocardiographic findings, patients were categorized into five subgroups: mild PS (n = 5), moderate PS (n = 45), severe PS (n = 112), critical PS (CPS, n = 8), and PS with supravalvular or subvalvular stenosis (n = 15). Minor cardiac structural abnormalities were also assessed. The CPS subgroup showed the highest frequency of minor anomalies, with all patients (8/8) presenting at least one abnormality. The proportions of patent foramen ovale (PFO), patent ductus arteriosus (PDA), and tricuspid regurgitation (TR) in this subgroup were 100%, 87.5%, and 75%, respectively, and 75% of patients had three concurrent anomalies. In comparison, the moderate PS subgroup showed the lowest frequency of minor anomalies (57.78%). The severe PS subgroup and the PS with supravalvular or subvalvular stenosis subgroup showed frequencies of 83.04% and 73.33%, respectively. The mild PS subgroup also showed a frequency of 100%, although the sample size was small (n = 5). Baseline clinical characteristics of each subgroup are summarized in Table 1.

**TABLE 1:** Baseline clinical characteristics of 185 PS patients.

Metric	Overall	Mild PS	Moderate PS	Severe PS	Critical PS	PS with supravalvular or subvalvular stenosis
Total number of patients, n (%)	185 (100.00)	5 (2.70)	45 (24.32)	112 (60.54)	8 (4.32)	15 (8.11)
Average age (years)^a^	1.25 ± 2.15	2.37 ± 1.94	1.87 ± 3.16	0.99 ± 1.49	0.64 ± 1.23	1.31 ± 2.45
Number of males, n (%)	95 (51.35)	2 (40.00)	21 (46.67)	57 (50.89)	5 (62.50)	10 (66.67)
Number of females, n (%)	90 (48.65)	3 (60.00)	24 (53.33)	55 (49.11)	3 (37.50)	5 (33.33)
Average flow velocity (m/s)^b^	4.54 ± 0.89	2.64 ± 0.20	3.60 ± 0.30	5.02 ± 0.64	4.71 ± 0.52	4.39 ± 0.79^c^
Minor cardiac anomalies, n (%)	143 (77.30)	5 (100.00)	26 (57.78)	93 (83.04)	8 (100.00)	11 (73.33)
Patients with PFO, n (%)	84 (45.41)	0 (0.00)	10 (22.22)	62 (55.36)	8 (100.00)	4 (26.67)
Patients with ASD, n (%)	60 (32.43)	5 (100.00)	15 (33.33)	32 (28.57)	0 (0.00)	8 (53.33)
Patients with PDA, n (%)	52 (28.11)	3 (60.00)	10 (22.22)	31 (27.68)	7 (87.50)	1 (6.67)
Patients with TR, n (%)	33 (17.84)	1 (20.00)	4 (8.89)	22 (19.64)	6 (75.00)	0 (0.00)
1 anomaly, n (%)	79 (42.70)	2 (40.00)	15 (33.33)	51 (45.54)	1 (12.50)	10 (66.67)
2 anomalies, n (%)	42 (22.70)	2 (40.00)	9 (20.00)	30 (26.79)	1 (12.50)	0 (0.00)
3 anomalies, n (%)	22 (11.89)	1 (20.00)	2 (4.44)	12 (10.71)	6 (75.00)	1 (6.67)

PS: pulmonary stenosis, PFO: patent foramen ovale, ASD: atrial septal defect, PDA: patent ductus arteriosus, TR: tricuspid regurgitation.

a The age of each patient was calculated by determining the number of days between the ultrasound diagnosis date and the date of birth, then dividing by 365.25 to convert into years.

b Multiple imputation was performed to impute the missing data for ten patients.

c Flow velocity at the narrowest point above or below the valve.

### 3.2 Summary of probe set alignment and variant filtering

**FIGURE 1:**
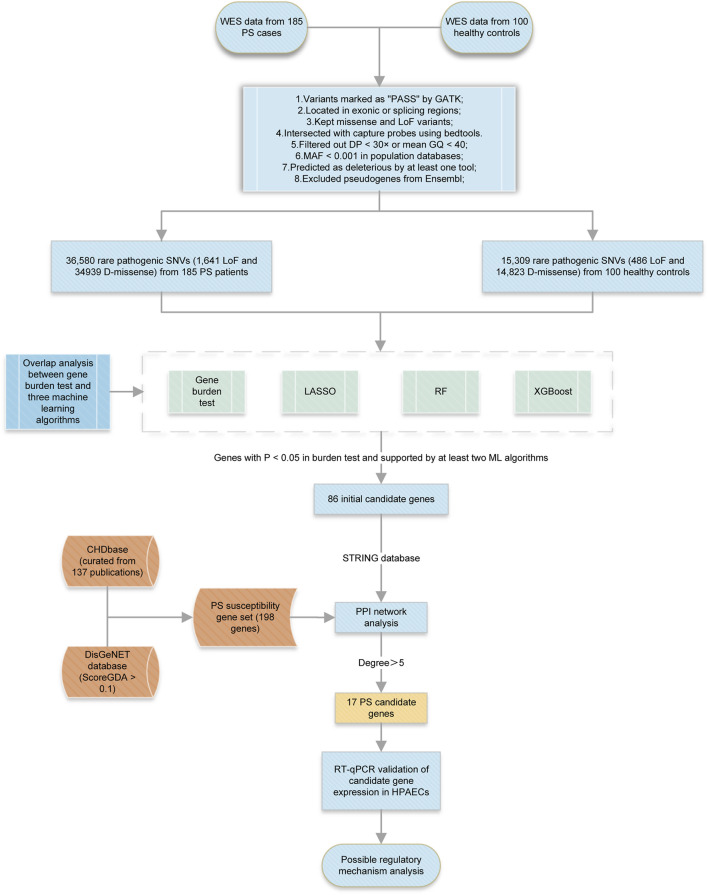
Schematic overview of the WES data analysis for PS at the gene level. This diagram outlines the analytical workflow used in the WES analysis. After pathogenicity-based variant filtering, candidate genes were prioritized through gene burden analysis in combination with machine learning algorithms. PPI network analysis was then performed, followed by measurement of gene expression levels in normal HPAECs. WES, whole-exome sequencing; PS, pulmonary stenosis; GATK, Genome Analysis Toolkit; LOF, loss-of-function; DP, depth of coverage; GQ, genotype quality; MAF, minor allele frequency; SNV, single nucleotide variant; LASSO, least absolute shrinkage and selection operator; RF, random forest; XGBoost, extreme gradient boosting; ML, machine learning; PPI, protein–protein interaction; RT-qPCR, reverse transcription quantitative polymerase chain reaction; HPAECs, human pulmonary artery endothelial cells.

**FIGURE 2:**
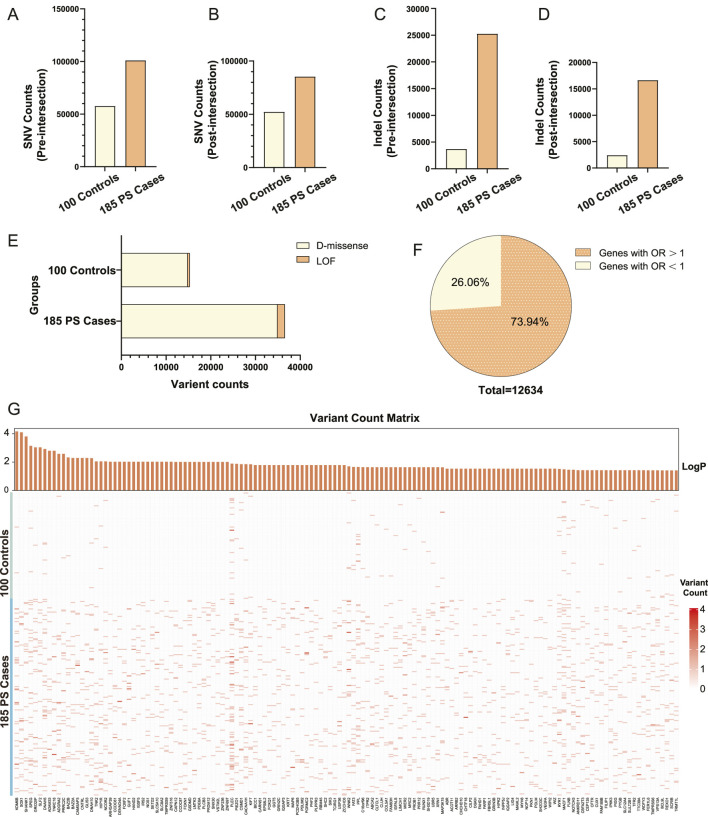
Variant screening and variant count matrix. **(A)** Comparison of SNV counts between the PS and control groups after initial filtering. **(B)** Comparison of SNV counts following probe-capture panel intersection based on the initially filtered variants. **(C)** Comparison of indel counts between the two groups after initial filtering. **(D)** Comparison of indel counts after intersecting with the probe-capture panel. **(E)** Comparison of rare damaging variants between the two groups, including deleterious missense and LOF variants. **(F)** Summary of gene-level variant burden in the PS group, highlighting genes with odds ratios (OR) greater than one or less than one compared to the control group. **(G)** Matrix of gene-level variant counts and corresponding p-values from the gene burden test. Fisher’s exact test was used to compare the PS and control groups. The upper bar plot displays p-values for 142 genes with p < 0.05, and the lower heatmap shows the distribution of variant counts across genes (x-axis) and samples (y-axis), with color intensity reflecting the mutation burden per gene.

A workflow was established to identify candidate pathogenic genes associated with PS (Figure 1). Following initial filtering, a total of 101,050 qualified nonsynonymous SNVs in exonic or splice-site regions were identified in 185 PS samples (average = 546 per individual), whereas 57,692 SNVs were identified in 100 controls under the same criteria (average = 577 per individual). After probe set alignment, both the total number and per-sample counts of SNVs remained comparable between the two groups (Figures 2A,B). For indels, 25,265 qualified variants (average = 137 per individual) were identified in the PS cohort, compared with 3,708 variants (average = 37 per individual) in controls. After probe set alignment, 16,632 indels remained in PS and 2,438 in controls (Figures 2C,D). Given the observed differences in indel counts between groups, subsequent analyses were restricted to SNVs. After applying depth of coverage and genotype quality thresholds, together with population frequency filtering and pathogenicity prediction, 36,580 rare pathogenic SNVs were retained in the PS cohort, including 34,939 deleterious missense variants and 1,641 LOF variants (141 splice-site and 1,500 stop-gain). Using the same criteria, 15,309 rare pathogenic SNVs were identified in controls, including 14,823 deleterious missense variants and 486 LOF variants (68 splice-site and 418 stop-gain) (Figure 2E).

### 3.3 Gene burden test

Compared with controls, 9,342 genes showed an OR > 1 in PS, accounting for 73.94% of the 12,634 analyzed genes (Figure 2F). Due to limited sample size and low variant counts, no genes remained significant after multiple testing correction (Benjamini–Hochberg method). Using a nominal threshold of P < 0.05, 142 genes were retained, including 46 genes with P < 0.01. Detailed results of the burden test and the mutation matrix are provided in Supplementary Table S5; Figure 2G.

### 3.4 Validation of rare-variant association using SKAT-O

To assess the consistency of the burden test results across analytical methods, we performed SKAT-O analyses on the same rare variant matrix. In total, 160 genes showed nominal significance (P < 0.05) in SKAT-O. Among the 142 genes identified by Fisher’s exact test (P < 0.05), 126 were also detected by SKAT-O, corresponding to an overlap of 88.73% (126/142). This overlap indicates concordance between the two approaches and suggests general consistency across different rare-variant association methods. While this does not constitute independent validation, it reduces the likelihood that the findings are driven solely by a single analytical framework. Detailed SKAT-O results are provided in Supplementary Table S6.

### 3.5 Prioritization of candidate genes using multiple machine learning algorithms

**FIGURE 3:**
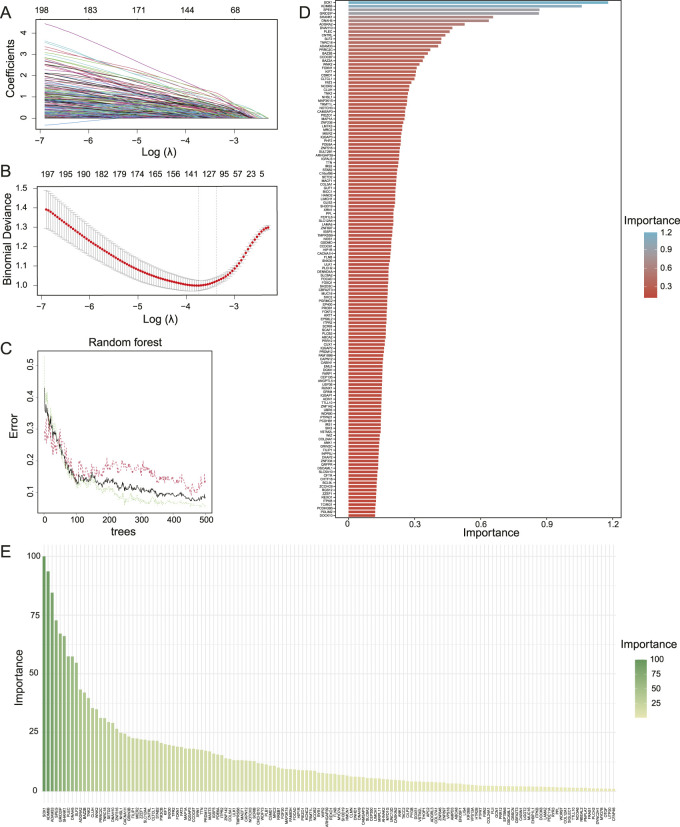
Candidate gene selection for PS using three machine learning algorithms. **(A)** Coefficient path plot from the LASSO model. The x-axis represents the logarithm of the regularization parameter λ, while the y-axis shows the coefficient values for each variable (gene) in the model. When λ is large, a stronger penalty is imposed, shrinking most coefficients toward zero. As λ decreases, the regularization effect weakens, allowing more variables to enter the model with non-zero coefficients. **(B)** Cross-validation error curve in the LASSO model. The x-axis corresponds to the log-transformed λ, and the y-axis indicates the binomial deviance, a measure of model error in classification tasks. The left vertical dashed line marks the value of λ that yields the minimum cross-validated error (lambda.min), while the right dashed line denotes the largest λ within one standard error of the minimum (lambda.1se). In this study, non-zero coefficients at lambda. min were selected as candidate predictors. **(C)** Out-of-bag (OOB) error plot from the random forest model. The x-axis shows the number of decision trees, and the y-axis represents the OOB error rate. The solid black line indicates the overall model error, while the red and green dashed lines show the OOB error for the case and control groups, respectively. As the number of trees increases, the error rate decreases and eventually stabilizes. The number of trees corresponding to the lowest error is considered the optimal number of estimators. **(D)** Variable importance plot from the random forest model. Feature importance was calculated based on the mean decrease in Gini impurity. The x-axis shows the importance scores, and the y-axis lists the gene names. The top 142 genes ranked by importance are displayed. **(E)** Feature importance plot from the XGBoost model. Feature importance was calculated based on gain. The x-axis shows the gene names, and the y-axis displays the corresponding importance scores. The top 142 genes ranked by importance are presented.

Since the burden test considers only the number of mutated versus non-mutated individuals, cumulative pathogenic variant counts were calculated for each individual across genes with OR > 1 to capture additional variation patterns. Based on this mutation count matrix, LASSO regression was performed using 10-fold cross-validation to identify the optimal model. The minimum λ value was 0.02374704, and the corresponding model retained 133 genes as candidate predictors (Figures 3A,B). For the RF algorithm, the optimal number of trees was determined to be 453, at which the classification error rate reached its minimum. The RF model was then constructed, and feature importance scores were calculated and used to rank genes (Figures 3C,D). For XGBoost, 10-fold cross-validation with four repeats was applied. Genes with importance scores greater than zero were retained, resulting in 154 genes (Figure 3E). Detailed results from all three algorithms are provided in Supplementary Table S7.

### 3.6 Comparison of gene sets from gene burden test and machine learning algorithms

**FIGURE 4:**
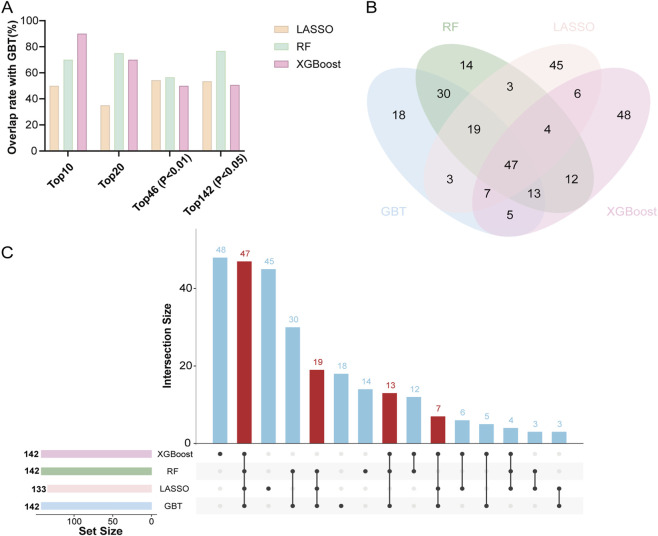
Overlap comparison of three machine learning algorithms versus burden test results and initial candidate gene selection. **(A)** Comparison of the overlap rates between machine learning algorithms and burden test at different levels. Two levels of overlap were assessed: (i) top-ranked genes (top 10 and top 20) and (ii) genes meeting global significance thresholds (p < 0.01 and p < 0.05). **(B)** Venn diagram illustrating the intersection of candidate genes identified by the four analytical approaches. **(C)** UpSet plot showing the detailed intersection patterns among the four algorithms. Red bars indicate the genes included as initial candidate genes in this study.

Using the burden test as a reference, overlap with each machine learning method was evaluated across different ranking thresholds (Figure 4A). Among the top 10 genes, XGBoost showed the highest overlap with the burden test, sharing 9 genes (*SOX1, KDM6B, ADAM33, SPEG, GRID2IP, SHANK1, DNAH5, SLF2,* and *ADGRA2*). RF shared 7 genes (*SOX1, KDM6B, SPEG, GRID2IP, SHANK1, DNAH5,* and *ADGRA2*), including complete overlap for the top 5 genes. LASSO showed 5 overlapping genes (*ADAM33, SLF2, SHANK1, SOX1,* and *GRID2IP*). Among the top 20 genes, overlap proportions were 35% (7/20) for LASSO, 75% (15/20) for RF, and 70% (14/20) for XGBoost. For the 46 genes with P < 0.01 in the burden test, RF overlapped with 26 genes (56.52%), LASSO with 25 (54.35%), and XGBoost with 23 (50%). Among the 142 genes with P < 0.05, RF showed 109 overlapping genes (76.76%), whereas XGBoost showed 72 (50.70%). As LASSO identified 133 genes, comparison with the 142 burden genes resulted in 76 overlaps (53.52%). Overall, RF showed higher overlap with the burden test across multiple thresholds. LASSO yielded fewer overlapping top-ranked genes but identified a more restricted feature set. XGBoost showed higher overlap among top-ranked genes, while overlap at broader thresholds was comparatively lower.

### 3.7 Selection of intersection genes

The burden test was used as the primary filtering approach, with LASSO, RF, and XGBoost applied as complementary methods for candidate gene prioritization (Figures 4B,C). Among the 142 genes identified by the burden test (P < 0.05), 47 were shared across all three machine learning algorithms. In addition, 19 genes were jointly identified by LASSO and RF, 13 by RF and XGBoost, and 7 by LASSO and XGBoost. In total, 86 genes were retained as preliminary PS candidate genes (Supplementary Table S8).

### 3.8 PPI network analysis and external dataset validation

**FIGURE 5:**
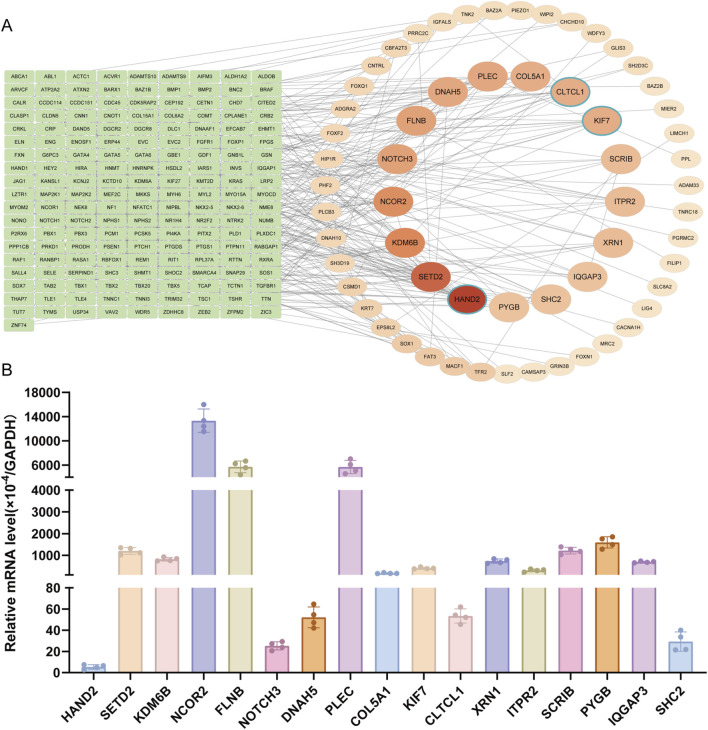
Screening of candidate genes and their mRNA expression in HPAECs. **(A)** PPI network analysis. This diagram depicts interactions between candidate genes and previously reported PS susceptibility genes. Genes within green rectangular boxes are known PS-related genes, while genes within orange-to-red gradient ellipses are the candidate genes identified in this study. Darker red indicates higher node degree. Genes marked with blue-bordered ellipses are candidate genes that have also been previously reported as PS-associated. **(B)** Bar plot of mRNA expression levels. The relative mRNA expression of the final candidate genes in normal HPAECs is shown, normalized to the internal control gene (GAPDH). Error bars represent standard deviation (SD) from four independent experiments (n = 4). This figure is presented for descriptive purposes only, and no statistical significance testing was performed.

PPI analysis was performed between the 86 candidate genes and previously reported PS-associated genes. Genes showing direct or indirect connections to known PS-associated genes were retained, and node degree was used to assess network connectivity. This analysis identified 60 genes with direct or indirect connections to known PS-associated genes (Figure 5A; Supplementary Table S9). The degree distribution of the network was right-skewed, with a median of 3 and a mean of approximately 5. Based on this distribution, a threshold of degree > 5 was applied, resulting in 17 genes retained as core candidate genes: *HAND2, SETD2, KDM6B, NCOR2, FLNB, NOTCH3, DNAH5, PLEC, COL5A1, KIF7, CLTCL1, XRN1, ITPR2, SCRIB, PYGB, IQGAP3,* and *SHC2*.

Among these, seven genes (*HAND2, SETD2, NOTCH3, DNAH5, COL5A1, KIF7,* and *CLTCL1*) have been previously reported as CHD-associated genes. In addition, *HAND2, KIF7,* and *CLTCL1* are included in the curated PS-associated gene set.

### 3.9 Expression of candidate genes in normal HPAECs

RT–qPCR analysis showed that all 17 candidate genes were detectable at the mRNA level in HPAECs (Figure 5B). Among them, *NCOR2* showed the highest relative expression compared with the internal control (*GAPDH*). Raw RT–qPCR data are provided in Supplementary Table S10.

## 4 Discussion

Although PS is a relatively common congenital heart disease, its genetic etiology and underlying mechanisms remain incompletely understood. In this study, peripheral blood samples from 185 sporadic PS patients and 100 healthy controls were analyzed using WES, and rare pathogenic SNVs were identified through multiple pathogenicity-filtering strategies. Given that sequencing data were derived from different sources, potential technical variability was addressed using a unified bioinformatic pipeline, restriction to intersected capture regions, and consistent filtering criteria. Under these conditions, no apparent global differences in variant count distributions were observed between groups. In addition, previous studies have reported an increased mutational burden in affected individuals (Shi et al., 2018), suggesting that the enrichment of SNVs observed in patients may reflect underlying biological variation rather than technical bias.

In this study, we focused on rare variants because they are more likely to exert larger functional effects (Cirulli and Goldstein, 2010; Tennessen et al., 2012) and may collectively contribute to disease susceptibility at the gene level (Wang et al., 2021), whereas common variants typically have smaller effects and require larger sample sizes to detect (Manolio et al., 2009). Accordingly, gene-level aggregation was used to evaluate the cumulative burden of rare variants (MAF < 0.001). The resulting gene set reflects statistical enrichment within this dataset and should be interpreted as candidate signals rather than evidence of causality, within a hypothesis-generating framework.

To assess analytical consistency, we compared Fisher’s exact test and SKAT-O results. Despite the lack of covariate adjustment in SKAT-O due to limited control metadata, 88.73% of genes identified by Fisher’s exact test were also detected by SKAT-O, and all 17 core candidate genes were retained. Although this overlap does not constitute independent validation, it suggests that the observed signals are not restricted to a single analytical method.

Machine learning approaches (LASSO, RF, and XGBoost) were applied for feature ranking and candidate prioritization. Among these, RF showed relatively higher concordance with burden test across multiple thresholds. Each method has inherent limitations: burden test may yield limited findings after multiple testing correction; LASSO selects a relatively small feature set; and RF/XGBoost rely on empirically defined importance thresholds. It should be noted that cross-method overlap was used as a pragmatic prioritization criterion rather than evidence of biological relevance. Using this strategy, 86 preliminary candidate genes were identified, and subsequent PPI analysis incorporating known PS-associated genes yielded 17 core candidates.

RT–qPCR analysis showed that all 17 genes are expressed in HPAECs, indicating transcriptional activity in a disease-relevant cell type. However, this does not imply disease specificity or functional involvement and should not be considered validation in the absence of case–control or tissue-specific evidence.

Among all 17 candidate genes, *HAND2*, *KIF7*, and *CLTCL1* have been previously proposed as PS susceptibility genes in the CHDbase database. In our network analysis, *HAND2* ranked highest by degree, and loss-of-function mutations have been implicated in familial PS (Sun et al., 2016). Of the remaining genes, *SETD2*, *NOTCH3*, *DNAH5*, and *COL5A1* are included in CHD candidate gene sets. The other genes, although not previously linked to PS, have evidence implicating them in cardiac development. Based on their biological functions, these genes can be grouped into epigenetic/transcriptional regulation, cytoskeletal/structural proteins, signaling pathways, and metabolism.

In epigenetic/transcriptional regulation, *KDM6B*, *NCOR2*, and *XRN1* may influence cardiac development through transcriptional and RNA metabolic processes. *KDM6B* promotes transcription via H3K27me3 demethylation (Long et al., 2020); its deficiency impairs cardiomyocyte differentiation and affects ventricular development (Akerberg et al., 2017). *NCOR2* encodes a transcriptional co-repressor, and knockout mice exhibit embryonic lethality with cardiac defects (Mottis et al., 2013), while combined *NCOR1*/*NCOR2* deletion causes somite abnormalities and cardiac dilation (Kumar et al., 2016). *XRN1* is essential for early embryogenesis, with knockout models showing severe developmental defects (Takaoka et al., 2021).

In cytoskeletal/structural proteins, *FLNB*, *PLEC*, and *IQGAP3* are involved in cellular architecture and vascular development. Loss of *FLNB* impairs endothelial migration and disrupts microvascular formation (Del Valle-Pérez et al., 2010; Zhou et al., 2007). *PLEC* localizes to cardiomyocyte intercalated discs and regulates WNT signaling (Yin et al., 2021), with variants associated with cardiomyopathy and arrhythmias (Thorolfsdottir et al., 2017; Vahidnezhad et al., 2022). *IQGAP3* regulates cytoskeletal organization and cell signaling, and its loss affects cell proliferation and migration (Fang et al., 2015).

In signaling pathways, *SCRIB*, *ITPR2*, and *SHC2* contribute to cardiac morphogenesis. *SCRIB* deficiency leads to ventricular defects (Boczonadi et al., 2014). *ITPR2* is required for cardiac development, with combined deletion resulting in embryonic lethality (Uchida et al., 2010). *SHC2* participates in VEGF signaling, promoting endothelial proliferation and angiogenesis (Ratcliffe et al., 2002; Wang et al., 2024).

In metabolism, *PYGB* encodes glycogen phosphorylase, which is expressed in cardiomyocytes, participates in glycogen metabolism, and has been proposed as a biomarker of myocardial ischemia (Yang et al., 2024).

Collectively, these genes are involved in WNT, NOTCH, VEGF, and epigenetic regulatory pathways. They may contribute to pulmonary valve and right ventricular development through effects on cardiomyocyte differentiation, endothelial function, and vascular development, although these associations require further validation.

Several limitations should be acknowledged. First, the relatively small sample size may limit statistical power for detecting rare-variant associations, and the absence of an independent replication cohort requires validation in larger and more diverse populations. Second, machine learning methods were used for feature ranking rather than predictive modeling, and their performance remains dependent on the available data and requires external validation. Third, this study focused on rare coding variants, whereas non-coding regulatory variants and other forms of genetic variation were not assessed and may also contribute to PS susceptibility.

In summary, by integrating gene-level burden test with multiple machine learning approaches, we identified candidate genes potentially associated with PS, including several not previously reported. These findings represent statistically prioritized candidates within this dataset. Further validation in independent cohorts and functional studies will be required to clarify their roles in PS pathogenesis.

## Data Availability

The datasets presented in this article are not readily available because of ethical and privacy restrictions. Requests to access the datasets should be directed to the corresponding authors.
